# A Retrospective Case Review of Firework-Related Ocular Injuries in Malaysia

**DOI:** 10.7759/cureus.74793

**Published:** 2024-11-29

**Authors:** Lhacha Wangdi, Niki Ho Wai Wye, Kamala D Lingam, Wong J Shyan, Abbas Hamid, Andrea L Barr, Maizan Yaakub, Nur F Azhani, Norhayaty Samsudin, Manoharan Shunmugam, Tajunisah Iqbal, Mae-Lynn Catherine Bastion

**Affiliations:** 1 Ophthalmology, Gyalyum Kelzang Choeden Wangchuck National Eye Centre, Thimphu, BTN; 2 Ophthalmology, Hospital Universiti Kebangsaan Malaysia, Kuala Lumpur, MYS; 3 Ophthalmology, International Specialist Eye Centre, Kuala Lumpur, MYS; 4 Ophthalmology, Hospital Tengku Ampuan Rahimah, Klang, MYS; 5 Ophthalmology, Hospital Sultanah Nur Zahirah, Kuala Terengganu, MYS; 6 Ophthalmology, Hospital Kemaman, Kuala Terengganu, MYS; 7 Ophthalmology, Oasis Eye Centre, Kuala Lumpur, MYS; 8 Ophthalmology, Faculty of Medicine, University of Malaya Eye Research Centre, Universiti Malaya, Kuala Lumpur, MYS; 9 Ophthalmology, Universiti Kebangsaan Malaysia Medical Centre, Kuala Lumpur, MYS

**Keywords:** closed globe injury, eye injury, eye laceration, fireworks, ocular injury, open globe injury, ruptured globe

## Abstract

Background: Fireworks are known to cause severe ocular injuries. This study was intended to examine the pattern and visual outcomes of firework-related severe eye injuries in Malaysia.

Methods: A retrospective review of fireworks-related eye injuries was conducted in Malaysia from 1^st^ July 2022 to 30^th^ June 2023. The data were collected from the ophthalmologists who treated and managed those cases. An open invitation was sent to the members of an ophthalmological society requesting case reports of severe ocular injuries requiring admission for further management.

Results: A total of 24 eyes were severely injured due to fireworks. Injuries were mostly among males, accounting for up to 66.67% (n=16), and children under 12 years old (33.33% (n=8)). Among the patients who were admitted due to severe fireworks ocular injuries, 58.33% (n=14) of them had open globe injuries followed by closed globe injuries (41.67% (n=10)), and eyelid injuries (41.67% (n=10)). Following firework eye injuries, at least 41.67% (n=10) of the eyes were blinded.

Conclusion: This study revealed severe eye injuries due to fireworks, ending in blindness in over one-third of the cases, and children were particularly vulnerable. Sight loss due to fireworks can be prevented through strong government policy, public awareness, and timely management.

## Introduction

Fireworks are mainly used as a means of celebration in different traditional festivals across the globe. Burning and blasting of fireworks are commonly seen during the celebration of New Year’s Eve. In addition, each country has traditional festivals, such as the Chinese New Year in China, Diwali in India, Aidil Fitri in Muslim countries, the Fourth of July in the United States, and the last Wednesday of the Persian year in Iran [1]. Malaysia is a racially diverse country; many festivals are celebrated together, such as New Year’s Eve, Independence Day, the Muslim Aidil Fitri festival, Chinese New Year, and Diwali. The loud sounds of the fireworks during these festivals are a testament to the widespread use of fireworks, in some neighborhoods beginning during the wee hours of midnight and extending beyond.

A series on firework-related injuries was published over 10 years ago in 2011, highlighting the impact of fireworks on people living on the east coast of Malaysia [1]. In this article, as many as 70% of those burnt by fireworks were children. Overwhelmingly, there was no eye protection at the time of injury, resulting in almost one in 10 persons blinded in the affected eye or eyes. This paper recommends health education, public awareness, and tighter legislation to protect the public. Since then, there has been no review of the impact of this favorite means of celebration in Malaysia and no data from the west coast of the country. Furthermore, our observation is that fireworks displays have become more spectacular, colorful, and loud, so to keep up with the audience, they need to be more dazzled with each display. However, there has been no monitoring at all as to whether the substances used to create these “improved” displays in recent years contain chemicals that may harm the environment or the public in the short or long term. More importantly, injuries from fireworks surely rank as a preventable cause of blindness. Hence, whether more recent types used are associated with an increase in ocular morbidity in our population, or whether conversely, previous experience has translated to improved care and prevention.

The use of fireworks in Malaysia has recently been legalized, and its usage has expanded across the country, especially during festivals. There are several unreported incidences of eye injuries related to fireworks in Malaysia, and the cases are expected to increase due to the legalization of fireworks in the country. Fireworks are known to cause devastating eye injuries leading to severe vision loss in those who are otherwise physically active, young males, and healthy persons [2]. The pattern of firework injury ranges from spontaneously healing conjunctival and corneal abrasions to open globe injury with severe posterior segment injuries, some of which require enucleation [3]. Eye injuries often result from poor judgment, rash stunts, or defects in manufacturing. Severe vision loss (visual acuity (VA) worse than 3/60) attributed to fireworks was reported to be mainly due to open globe injury and lens injuries [4]. Eye injuries and vision loss due to fireworks are preventable if proper public awareness and strong government policies on fireworks are in place. Here we report a series of firework-related severe eye injuries and their associated visual impact on the Malaysian population in recent times.

## Materials and methods

This study was conducted as a retrospective analysis of firework-related ocular injuries in Malaysia from 1^st^ July 2022 till 30^th^ June 2023. Prior to the study, ethical clearance was obtained from the Research Ethical Committee of the National University of Kebangsaan, Kuala Lumpur, Malaysia. The objectives of this study were to analyze the demographic profile, visual impact, and severity of firework-related ocular injuries in Malaysia. Cases were compiled based on history sheets and clinical information recorded by ophthalmologists who managed firework-related eye injuries. The invitation was issued using the social media platform WhatsApp (Meta Platforms, Menlo Park, CA, USA) for telemedicine to Malaysian ophthalmologists from the Malaysian Society of Ophthalmology, who were in active clinical service and might have treated eye injuries due to fireworks previously. This series is compiled from the responses.

Inclusion criteria for the study were firework-related eye injuries requiring admission for further management. Cases without complete clinical information and lost follow-up cases were excluded. 

A convenience sampling method was used to collect the cases retrospectively. The case review included clinical history, initial assessment, relevant investigations, grading of eye injuries, definitive management, and management outcomes of fireworks-related eye injuries. In the clinical history, we reviewed the nature and types of fireworks that caused the eye injuries. The initial assessment mainly focused on the presenting VA and the detailed examination of the anterior and posterior segments of an eye. 

The severity of the eye injury was based on the VA upon presentation to the hospital. After the review of the initial assessment, firework-related eye injuries were graded based on the Birmingham Eye Trauma Terminology System (BETTS) [5]. As per the BETTS grading, eye injuries were classified as either open globe injuries or closed globe injuries. Open globe injuries were further classified as penetrating eye injuries, intraocular foreign bodies (IOFB), and perforating eye injuries or ruptured eyes. Close globe injuries were sub-classified as lamellar laceration or contusion.

Following the initial assessment and grading of eye injuries, we looked at both the medical and surgical management aspects. The outcome of the eye injury was based on the final best-corrected visual acuity (BCVA) of the injured eye. The impact of ocular injuries was based on the initial presenting VA, severity of injury based on BETTS classification [5], and final visual outcome after the treatment. Case data were compiled in a Microsoft Excel spreadsheet (Microsoft Corp., Redmond, WA, USA). Statistical analysis was done using IBM SPSS Statistics software, version 20 (IBM Corp., Armonk, NY, USA).

## Results

During the one-year retrospective case review, there were 24 reported incidences of firework-related eye injuries requiring admission for further management. The majority of the injuries were among males, accounting for up to 66.67% (n=16). While all the reported cases were aged 40 years and below, the most common age group affected was children (33.33% (n=8)). Most of the injuries were reported to have occurred during a playful celebration of the occasions. Eye injuries happened mostly due to the sudden, unexpected explosion of the fireworks while trying to light them. Due to the huge explosion and misdirection of the fireworks, 37.50% (n=9) of bystanders were injured. The most common fireworks causing severe eye injury in this case review were firework bombs (37.50% (n=9)), followed by bottle rockets (29.17% (n=7)), while the rest were sparklers and miscellaneous firecrackers. None of those patients reported the use of eye protectors during the fireworks incident. 

All the patients had undergone initial assessments, and they were provided with emergency care followed by further definitive management whenever necessary by their ophthalmologist. Initial assessment included recording presenting VA, intraocular pressure (IOP) wherever appropriate, slit lamp examination, and fundus examination. The investigations included gonioscopy unless contraindicated in penetrating or perforating eye injuries or when the view was poor, B-scan, optical coherence tomography (OCT), X-ray, or CT scan as and when necessary. Patients were initially managed in the outpatient department (OPD), followed by admission and management in the ward or referral to higher eye care centers based on the severity of eye injuries. In this study, all patients were admitted and managed inward due to severe ocular injuries.

Among the patients with fireworks-related eye injuries, 58.33% of them had open globe injuries. Penetrating ocular injury was the most common type of open globe injury, as in Table 1. The other common firework injuries were closed globe injuries and eyelid injuries, accounting for 41.67% (n=10) each. Firework-related eye injuries according to the BETTS classification are depicted in Table 1.

**Table 1:** Types of eye injuries among the study participants based on the Birmingham Eye Trauma Terminology System (BETTS) classification *number of eye injuries reported BETTS criteria source: [5]

S. No.	Type of firework-related eye injuries, % (n)	Classification as per the BETTS classification, % (n*)
1	Open globe injury: 58.33 (14)	1a) Penetrating injury: 33.33 (8)
		1b) Intraocular foreign bodies (IOFB): 12.50 (3)
		1c) Perforating injury: 0.00
		1d) Ruptured globe: 12.50 (3)
2	Closed globe Injury: 41.67 (10)	2a) Contusion: 41.67 (10)
		2b) Lamellar laceration: 0.00

**Figure 1:**
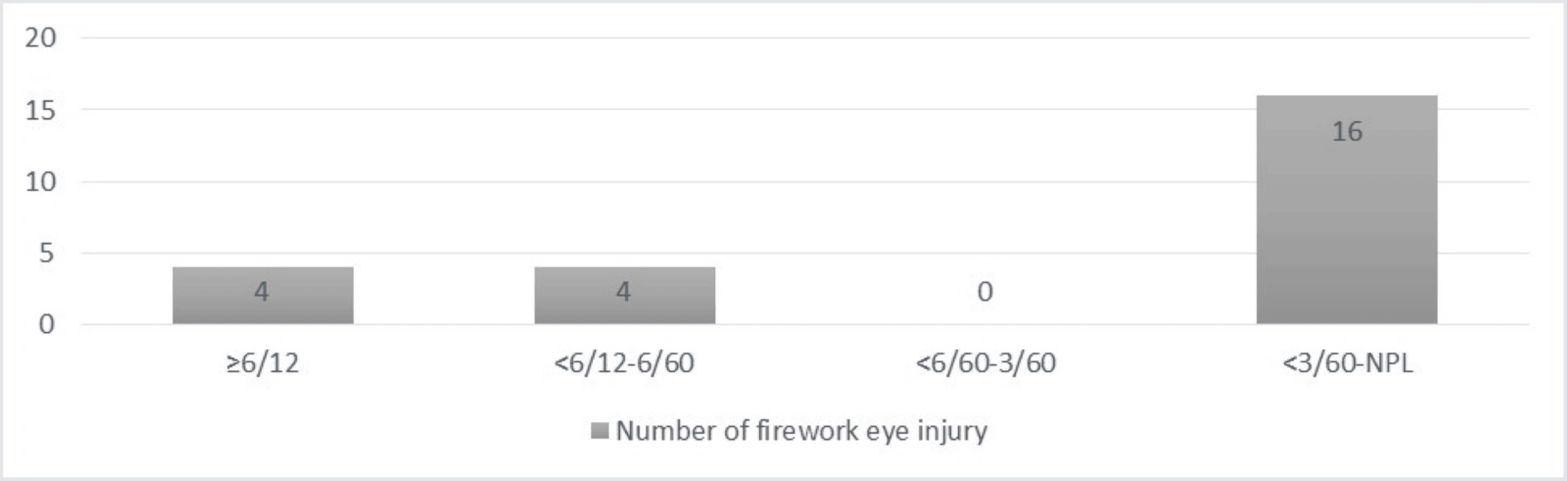
Visual acuity of the affected eyes based on the presenting visual acuity NPL: no perception of light

Based on presenting VA, 83.33% (n=20) out of 24 affected eyes had VA worse than normal, of which three of the affected eyes had VA of no perception of light (NPL) on presentation to the hospital (Figure 1). Worse eye injuries with presenting VA worse than hand motion (HM) were due to ruptured globes. 

Following treatment, 41.67% (n=10) had visual acuity worse than 3/60, out of which five ended up with VA of NPL due to severe injury. As a result of severe injury, two eyes were surgically removed, one each of eviscerated and enucleated (Table 2).

**Table 2:** Visual outcomes of firework-related eye injuries after the treatment based on injuries as per the BETTS criteria VA: visual acuity; n: number of injured eyes; NPL: no perception of light; IOFB: intraocular foreign bodies; BETTS: Birmingham Eye Trauma Terminology System

Type of firework-related eye injuries	VA ≥ 6/12, %(n)	VA<6/12-6/60, %(n)	VA <6/60-3/60, %(n)	VA <3/60-NPL, %(n)
Penetrating injury	25(6)	0.00	0.00	8.33 (2)
IOFB	8.33(2)	0.00	0.00	4.17(1)
Rapture	0.00	0.00	0.00	12.5(3)
Close globe injury	25(6)	0.00	0.00	16.67(4)
Total: % (n)	58.33% (14)	0.00	0.00	41.7% (10)

**Figure 2:**
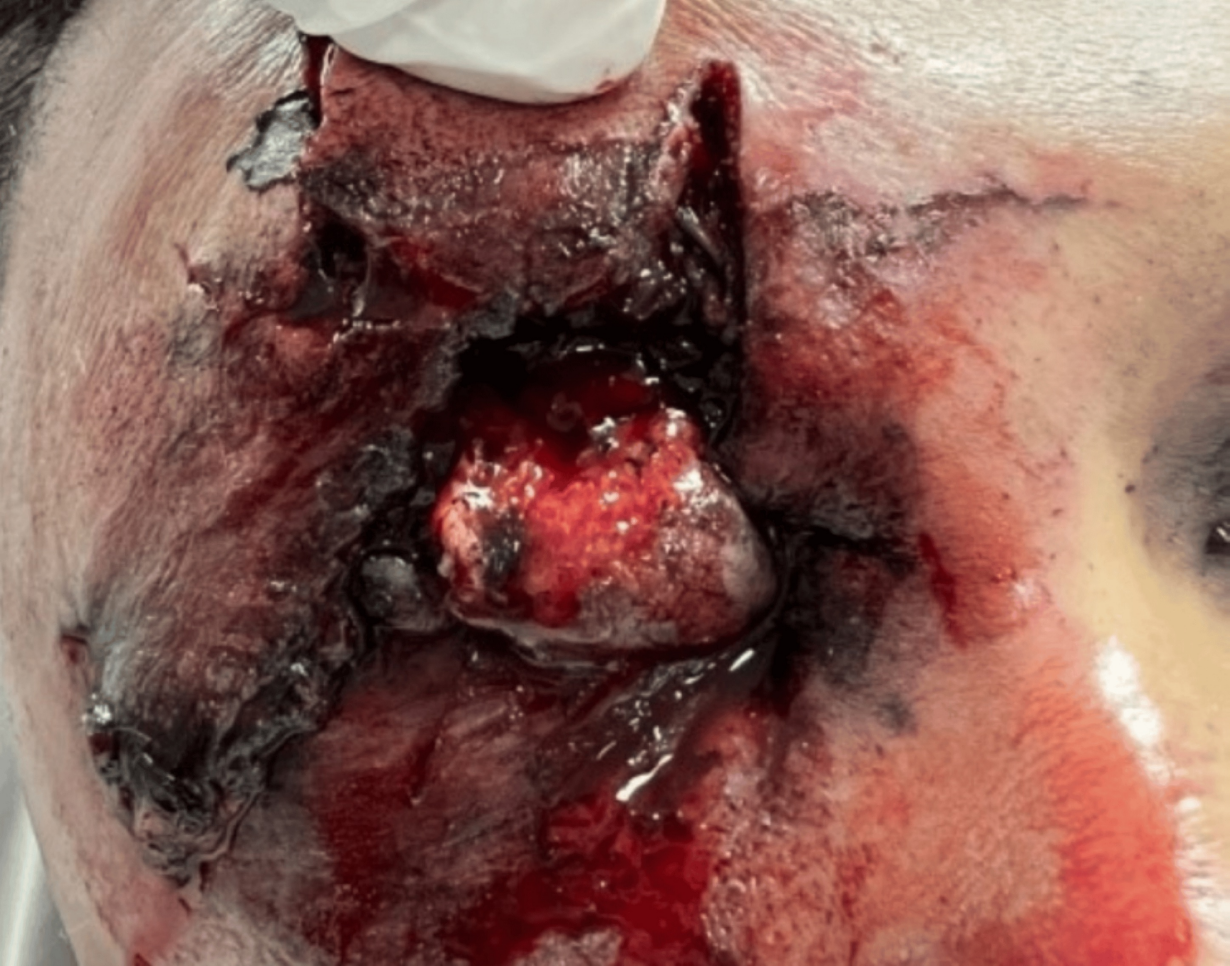
A 34-year-old male patient had a ruptured globe with extensive eyelid laceration following a blast injury from the firework. There was no visual potential as the eye was damaged beyond repair. The patient underwent enucleation.

**Figure 3:**
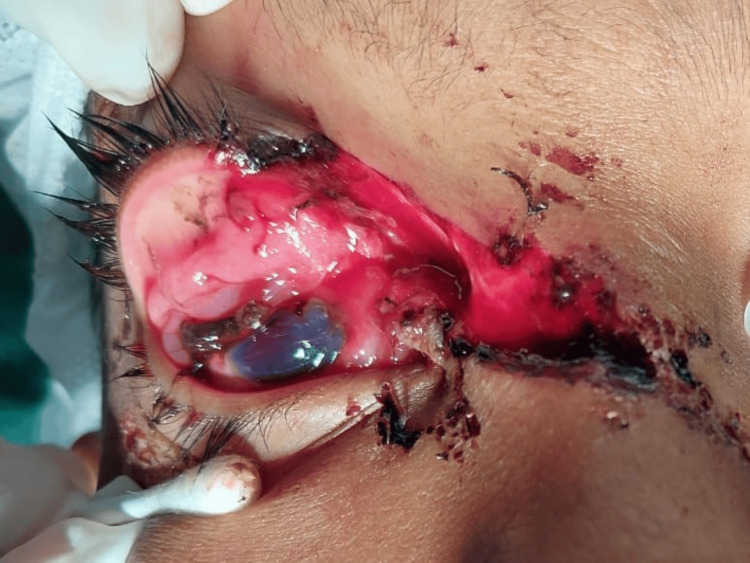
A nine-year-old boy had a severe right eye injury due to a firework explosion, a ruptured globe with uveal tissue prolapse, and an upper eyelid laceration extending over the nasal bridge. Primary repair was done; however, the patient’s visual acuity remained NPL. NPL: no perception of light

**Figure 4:**
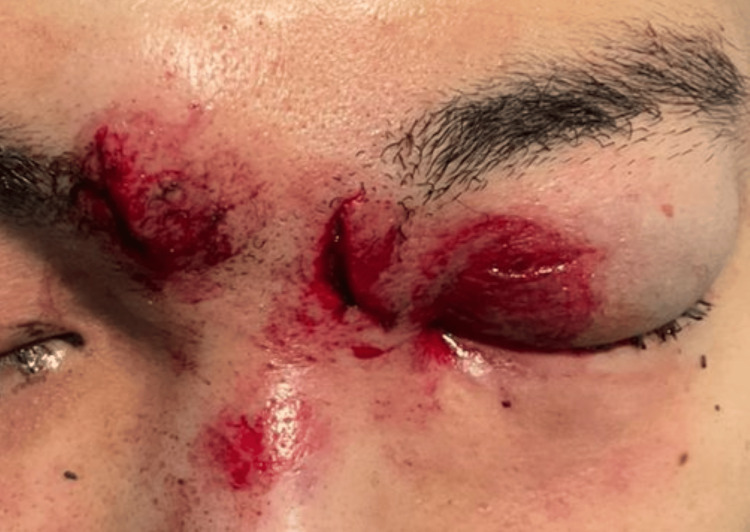
Clinical photograph of a 20-year-old male patient with an eyelid injury due to the explosion of a firework. Laceration of skin over the right eyebrow and left eyelid was noted. He also had concomitant blunt injury of the left eye; hyphema, angle recession, and commotio retinae. After treatment, visual acuity was 6/36.

The most devastating firework-related eye injuries reported were the ruptured globes. There were three cases of severe globe ruptures with no visual potential. Firework-related eye injuries are depicted in Figures 2-4.

## Discussion

Most firework-related ocular injuries are related to festivals such as New Year celebrations or local festivals [5-8]. Globally, fireworks are mostly lit by younger people; therefore, children and young male adults are most susceptible to fireworks injuries [2, 8]. While the use of fireworks is not illegal in Malaysia, unsupervised firework activity among children can lead to severe eye injuries and blindness. In this study, all the reported patients of firework-related eye injuries were aged 40 years and below, and the most affected age groups were school-going children between six and 12 years of age. This has not changed at all compared to more than 10 years ago [1]. Most of the eye injuries were a result of direct handling of fireworks; however, 37.5% of the injuries were bystanders, which is comparable with another previous study which was similar [4]. None of the patients reported the use of eye protectors during the accident, which implies there is no existing safety policy, showing a lack of response from the community to the previous reports [1]. This finding highlights the need for increasing public awareness regarding the fireworks health hazard and advocating the use of eye shields during fireworks play. The use of protective eye gear while handling firecrackers is very crucial, and it is known to prevent firework-related eye injuries [9].

Fireworks can blind a person because they cause a form of severe ocular burn injury. Fireworks are known to cause severe eye injuries and related visual impairment [10]. When ignited, they reach high temperatures that, once in contact with the delicate outer coats of the eyelid and eye, can cause disfigurement or blindness. Fireworks of the projectile type add another element to the mechanism of eye injury as they impact the eye at high speed, resulting in devastating internal injuries that are often irreparable, even by surgery, as illustrated by several cases from this series. Globally, the commonly reported eye injuries related to fireworks are corneal abrasion, hyphema, eyelid injuries, cornea-scleral laceration, vitreous hemorrhage, and a ruptured globe [11-13]. In this case series, the majority had open globe injuries, followed by closed globe injuries and eyelid injuries. This was comparable with previous similar studies [4, 13]. The higher incidence of open globe injuries related to fireworks was also reported in a study from China [8]. In this series, the most severe eye injuries were due to the ruptured globes, accounting for 12.5%, out of which two eyes had to undergo evisceration, reflecting the high ocular morbidity. 

Legislation and policy planning to monitor or restrict the use of fireworks during festivals may play an important role in preventing fireworks injuries [14]. For example, in Hawaii, there was a significant decrease in firework-related injuries where the legislation requiring permits for a specified number and type of fireworks and limiting access to persons 18 years and older was put in place [14]. Many countries around the world have banned consumer fireworks. India has also enjoyed the benefits of effective legislation around firework sales, reducing not only the incidence of trauma but also reducing air pollution [15]. Nevertheless, legislation without enforcement will also fail. During festivals, the blasting of fireworks continues even at midnight, and various types of fireworks are being used, including homemade miscellaneous fireworks, which could be more dangerous. Unsupervised use of fireworks among children is one of the greatest risks, as children are mostly unaware of the gravity of fireworks blasts. Poor public awareness about the health hazard and manufacturer default of fireworks are equally important risk factors for firework-related injuries. Hence the role of a responsible society is to keep its public well informed and set societal guidelines. Fireworks should therefore only be ignited by professionals. Professionals should wear eye protection when handling them and follow the instructions provided.

Visual outcome following firework injury is often reported as very poor despite providing appropriate management [13]. In this retrospective review, the majority of the injured eyes had VA worse than 3/60, and three eyes had a VA of NPL on presentation. Even with effective treatment, 41.67% of the injured eyes had poor post-treatment visual outcomes worse than 3/60, of which five eyes became NPL. The visual outcome in this series differs from a previous firework-related ocular injury case series reported from Malaysia in 2011, where only three eyes had VA worse than 1/60 and no eye became NPL [1]. This may be due to a development in the last 10 years in the type of fireworks that produce high explosions or increased usage of fireworks compared to before. The increasing trend of firework eye injury and poor visual outcomes was also reported by Unterlauft et al. in the year 2014 [7]. The aftermath of the fireworks eye injury and the poor visual outcomes highlight the grievous eye injuries due to fireworks. Firework-related eye injuries are not only potentially blinding, but they can also cause severe facial injuries leading to facial disfigurement. The fellow eye may, in rare instances, not be spared, either being involved during the firework-related injury or years later in a condition known as sympathetic ophthalmitis, requiring lifelong systemic immunosuppressive therapy [16].

This study was conducted retrospectively based on the response from the WhatsApp telemedicine group. The patients' identities were anonymized to prevent a breach of confidentiality, and verbal consents were obtained from the patients for the use of their clinical information for research purposes. Due to the retrospective nature of the study, the consents were obtained from the patients by phone call. Although the patients' identities and their medical information were kept confidential, the use of WhatsApp and telephone was not the specialized platform; therefore, an appropriate and secure healthcare-compliant platform is needed for clinical data transmission for future studies. 

This case analysis included only severe eye injury cases which were admitted and managed in the hospital. The overall findings in this study may not be a true representation of firework-related eye injuries in Malaysia. Further large-scale, prospective surveys on firework-related eye injuries are needed to get a clear picture of the pattern of ocular injury and the visual impact of firework-related eye injuries in Malaysia. 

## Conclusions

The number of firework-related eye injuries and blindness will continue to rise unless strong local policies are put in place to restrict or monitor fireworks in the country. Policies such as restricting the timeframe of firework usage, monitoring the types of fireworks, and using eye protectors during firework play may help in reducing firework-related eye injuries. Public awareness about the health hazards related to fireworks and the supervision of children’s play are equally important. While early recognition and timely management of eye injuries are very important, it is always better to prevent injury itself because eye injuries due to fireworks are mostly severe and sight-threatening in a very significant number. 
